# Extracorporeal Cardiopulmonary Resuscitation for Perioperative Cardiac Arrest in Noncardiac Surgery: A Nationwide Cohort Study in Japan

**DOI:** 10.1097/ao9.0000000000000013

**Published:** 2026-04-15

**Authors:** Yoshitaka Aoki, Yuki Shiko, Yohei Kawasaki, Mikio Nakajima, Kenya Oda, Koki Ubukata, Yusuke Mizobuchi, Satoshi Naruse, Yuji Suzuki, Kensuke Kobayashi, Hiromi Kato, Soichiro Mimuro, Yoshiki Nakajima

**Affiliations:** 1 Yoshitaka Aoki, M.D., Ph.D., Kenya Oda, M.D., Koki Ubukata, M.D., Yusuke Mizobuchi, M.D., Ph.D., Yuji Suzuki, M.D., Kensuke Kobayashi, M.D., Ph.D., Hiromi Kato, M.D., Ph.D., Soichiro Mimuro, M.D., Ph.D., and Yoshiki Nakajima, M.D., Ph.D.: Department of Anesthesiology and Intensive Care Medicine, Hamamatsu University School of Medicine, Hamamatsu, Shizuoka, Japan.; 2 Yuki Shiko, Ph.D., and Yohei Kawasaki, Ph.D.: Department of Biostatistics, Graduate School of Medicine, Saitama Medical University, Saitama, Japan.; 3 Mikio Nakajima, M.D., M.P.H., Ph.D.: Emergency and Critical Care Center, Tokyo Metropolitan Hiroo Hospital, Shibuya-ku, Tokyo, Japan; Department of Clinical Epidemiology and Health Economics, School of Public Health, The University of Tokyo, Bunkyo-ku, Tokyo, Japan.; 4 Satoshi Naruse, M.D.: Perinatal Center, Hamamatsu University School of Medicine, Hamamatsu, Shizuoka, Japan.

## Abstract

**Background:**

Perioperative cardiac arrest is rare but often catastrophic. The use of extracorporeal cardiopulmonary resuscitation (ECPR) with veno-arterial extracorporeal membrane oxygenation in perioperative settings remains limited. This study evaluated the characteristics and outcomes of patients who underwent ECPR for perioperative cardiac arrest.

**Methods:**

This retrospective cohort study used data from the Japanese Intensive Care PAtient Database, a nationwide registry of 129 intensive care units (ICUs), covering fiscal years 2015 to 2023. Adults undergoing noncardiac surgery who experienced cardiac arrest before ICU admission were included. Patients were classified as ECPR if veno-arterial extracorporeal membrane oxygenation was documented. The primary outcome was in-hospital mortality. Secondary outcomes were discharge to home, transfer to another facility, ICU mortality, length of hospital stay, and length of ICU stay. Multivariable logistic regression, adjusted for potential confounders, was used to assess the association between ECPR and in-hospital mortality.

**Results:**

Among 518 patients with perioperative cardiac arrest, 44 (8.5%) received ECPR. The mean age was 66 yr and 62% were male. Patients who received ECPR had higher severity scores, higher lactate levels, and more frequent use of mechanical ventilation and catecholamines than those without ECPR. Unadjusted in-hospital mortality was significantly higher in the ECPR group (61.4 *vs.* 34.0%; *P* = 0.001). In the multivariable logistic regression analysis, no statistically significant association was observed between ECPR and in-hospital mortality (odds ratio, 0.85; 95% CI, 0.37 to 1.97).

**Conclusions:**

In this nationwide cohort, ECPR was used in 8.5% of patients with perioperative cardiac arrest in noncardiac surgery. Its effect on in-hospital mortality in the studied population remains inconclusive due to the limited sample size. Further data accumulation is warranted to clarify the clinical role of ECPR in this setting.

**Editor’s Perspective**

**What We Already Know about This Topic**

- The feasibility, efficacy, and safety of extracorporeal cardiopulmonary resuscitation (ECPR) using veno-arterial extracorporeal membrane oxygenation (VA-ECMO) in patients with out-of-hospital cardiac arrest have been extensively studied
- When applied to highly selected patients—such as those with witnessed arrest, short no-flow time, shockable rhythm, and rapid cannulation in experienced centers—ECPR has been associated with favorable neurologic outcomes of approximately 20 to 40% in some trials and high-performing systems

**What This Article Tells Us That Is New**

- In Japan, ECPR was used in 8.5% of perioperative cardiac arrests among noncardiac surgical patients
- Unadjusted in-hospital mortality was higher in patients who received ECPR compared with those who received conventional CPR; however, patients treated with ECPR were more severely ill at baseline
- In the perioperative setting for noncardiac surgery, ECPR did not appear to be associated with additional harm during resuscitation

Perioperative cardiac arrest is a rare but life-threatening complication that requires immediate and effective intervention.^1,2^ Anesthesiologists must be prepared to initiate prompt, guideline-based cardiopulmonary resuscitation (CPR) and consider advanced strategies to optimize survival and neurological outcomes.^3–5^

Extracorporeal CPR (ECPR) involves the initiation of veno-arterial extracorporeal membrane oxygenation (VA-ECMO) during cardiac arrest to provide temporary circulatory support and serve as a bridge to definitive therapy. In cases of in-hospital and refractory out-of-hospital cardiac arrest of presumed cardiac origin, observational studies and registry data suggest that ECPR can improve survival and neurological outcomes,^6,7^ leading international clinical practice guidelines to recommend its use in carefully selected patients.^8,9^ In addition, a randomized controlled trial demonstrated improved survival with early extracorporeal membrane oxygenation (ECMO)-facilitated resuscitation in patients with refractory ventricular fibrillation out-of-hospital cardiac arrest.^10^ By contrast, survival rate after perioperative cardiac arrest is generally higher than after ward-based arrests,^11^ which may partly explain why ECPR is less frequently used in this setting. The 7th National Audit Project (NAP7) of the Royal College of Anaesthetists in the United Kingdom reported that ECPR was attempted in only 19 of 881 patients (2%) with perioperative cardiac arrest.^1^ A recent analysis of the Extracorporeal Life Support Organization registry—including periprocedural ECPR cases—demonstrated that in-hospital ECPR outcomes vary substantially by the location of cardiac arrest.^12^

In the perioperative setting, one guideline offers only a weak recommendation for ECPR,^3^ another suggests VA-ECMO for pulmonary embolism as a bridge to definitive treatment,^4^ whereas others make no mention of the intervention,^5^ reflecting the paucity of high-quality evidence. Most available studies are small, single-center series with heterogeneous surgical populations, which limits the generalizability of their findings.^13,14^ In cardiac surgery, VA-ECMO is often initiated for failure to wean from planned cardiopulmonary bypass, making it difficult to distinguish from ECPR performed for intraoperative cardiac arrest.^15^ This overlap contributes to the challenges of evaluating the effectiveness of ECPR. The survival benefit of ECPR in patients with perioperative cardiac arrest—particularly during noncardiac surgery—remains uncertain and represents an important clinical question for anesthesiologists worldwide.

The aim of the present study was to describe the baseline characteristics, intensive care unit (ICU) interventions, and clinical outcomes of these patients, with a specific focus on ECPR. We hypothesized that ECPR could improve the survival rate in noncardiac surgical patients experiencing perioperative cardiac arrest.

## Materials and Methods

This multicenter retrospective cohort study used clinical data prospectively collected in the Japan Intensive Care PAtient Database (JIPAD) for fiscal years 2015 to 2023 (April 2015 to March 2024). Ethical approval was obtained from the Institutional Review Board of Hamamatsu University School of Medicine (approval no. 25-156). The study protocol was also approved by the JIPAD Working Group before data provision. Because the dataset was fully anonymized, the requirement for informed consent was waived. The study was conducted in accordance with the Strengthening the Reporting of Observational Studies in Epidemiology guidelines.

### Database

Since 2014, the Japanese Society of Intensive Care Medicine has managed the JIPAD in accordance with international ICU database standards. At the time of ethics committee approval for this study, the registry had prospectively collected clinical data from 433,661 patients across 129 ICUs. Certified physicians at participating institutions, all engaged in daily ICU care, submit anonymized information on diagnoses, ICU admission routes, vital signs, treatment details, complications, and discharge outcomes. To ensure accuracy, physicians responsible for data management receive training to validate the completeness and reliability of entries. The JIPAD Working Group conducts continuous data reviews and provides feedback to each ICU on data quality. For research purposes, the group authorizes study protocols and supplies cleaned, anonymized datasets to investigators. A detailed description of JIPAD has been published previously.^16^

### Patients

We included patients who underwent noncardiac surgery and were admitted directly to the ICU from the operating room. Patients were excluded if they were younger than 16 yr or did not experience perioperative cardiac arrest. Perioperative cardiac arrest was defined as either a primary diagnosis of cardiac arrest or documentation of “cardiac arrest before ICU admission” accompanying another primary diagnosis. In JIPAD, cardiac arrest is defined as the absence of a palpable pulse or the need for chest compressions. Transient arrests that did not require CPR were excluded. Cardiac arrests before ICU admission included events occurring before entry into the operating room, during surgery, or in transit to the ICU. Following the approach of a previous study,^11^ patients who developed cardiac arrest after ICU admission were excluded. Surgical procedures were classified as elective or emergency according to the system used in our previous study.^17^ In the JIPAD, emergency surgery was defined as nonelective surgery performed before ICU admission or within 24 h of ICU admission.

### Exposure and Outcomes

The patients were divided into ECPR and conventional CPR groups based on the presence or absence of VA-ECMO in the dataset. The primary outcome was in-hospital mortality. The secondary outcomes were discharge to home, transfer to another healthcare facility, ICU mortality, length of hospital stay, and length of ICU stay.

### Definition of Critical Care Beds

Intensive care units eligible for participation in JIPAD were defined as distinct hospital units dedicated to the care of critically ill patients, equipped with essential medical infrastructure, and staffed according to national criteria for ICU or emergency and critical care unit management fees in Japan. These criteria require continuous physician presence, 24-h nursing coverage with a nurse-to-patient ratio of 1:2, and at least 15 m^2^ of dedicated space per bed. In addition, at least two full-time intensivists certified by the Japanese Society of Intensive Care Medicine must be employed, each devoting at least 80% of their working hours to the ICU. These standards establish the minimum requirements for JIPAD participation and reflect expectations for high-resource critical care environments.^18,19^

### Variables

Patient-level variables included age, sex, height, weight, and body mass index at admission, as well as whether the patient underwent emergency surgery and the number of days from hospital admission to ICU admission. Chronic comorbidities recorded were heart failure, respiratory failure, liver cirrhosis, acute leukemia, lymphoma, metastatic cancer, receipt of immunosuppressive therapy, and maintenance dialysis. ICU interventions and prognostic indicators within the first 24 h of ICU admission included use of mechanical ventilation, pulmonary artery catheterization, occurrence of acute kidney injury, and administration of catecholamines (dopamine, norepinephrine, and dobutamine). Severity scores—Acute Physiologic and Chronic Health Evaluation (APACHE) II and III, Simplified Acute Physiology Score II, Sequential Organ Failure Assessment, and Japan Risk of Death—were used to assess illness severity. The worst lactate level within 24 h of ICU admission was also recorded. The Japan Risk of Death score was employed for case-mix adjustment, given its superior predictive performance for in-hospital mortality compared with the APACHE III score in the JIPAD cohort.^20^ Surgical procedures were identified based on JIPAD diagnosis codes, all of which are listed in Supplemental Digital Content 1 (https://links.lww.com/ALNO/A6).

Hospital-level variables included hospital type (national university hospital, national hospital, private university hospital, private hospital, public university hospital, or public hospital), ICU functional type (emergency and medical-surgical ICU, medical-surgical ICU, emergency ICU, surgical ICU, or other units), ICU operational model (mandatory critical care consultation, closed ICU, or elective critical care consultation), total number of hospital beds, number of ICU beds, and number of full-time ICU staff.

### Statistical Analysis

Continuous variables are presented as mean ± SD or median (interquartile range), depending on their distribution, and categorical variables are presented as number (%). Between-group comparisons were conducted using Student’s *t* test for normally distributed continuous variables and the Wilcoxon rank-sum test for nonnormally distributed variables, while the chi-square test was used for categorical variables. To account for the clustering of patients within facilities, a multivariable logistic regression analysis using Generalized Estimating Equations was performed to evaluate the association between ECPR and in-hospital mortality. The primary exposure was the presence or absence of ECPR. Age, sex, APACHE III score, emergency surgery, and obesity (body mass index of 25 kg/m^2^ or greater) were selected *a priori* based on clinical relevance and previous literature, and all were entered simultaneously into the model (forced entry).^21,22^

Three sensitivity analyses were performed to ensure the robustness of the results. First, backward selection (removal criterion *P* > 0.05) was applied with ECPR forced to remain in the model, while all other covariates were subject to selection. Second, to address potential nonlinearity, continuous covariates were replaced with categorical variables: age was dichotomized (younger than 65 *vs.* 65 yr or older) and the APACHE III score was divided into two groups based on the median value. Analyses were performed on complete-case data. Third, to rigorously adjust for confounding by indication, a propensity score-matched analysis was conducted. Propensity scores were estimated using a logistic regression model incorporating the baseline characteristics listed in table 1, with the exception of height and weight to avoid multicollinearity. One-to-one nearest neighbor matching without replacement was performed using a caliper width of 0.2 of the SD of the logit of the propensity score.^23^ Covariate balance was assessed using absolute standardized differences, with a value of less than 10% considered to indicate negligible imbalance.^24^

**Table 1.** Baseline Characteristics

Variable	Overall (N = 518)	ECPR (n = 44)	Conventional CPR (n = 474)	*P* Value
Age, yr, mean ± SD	66.0 ± 16.6	62.8 ± 17.2	66.3 ± 16.5	0.18
Male sex, n (%)	319 (61.6)	25 (56.8)	294 (62.0)	0.50
Height, cm, mean ± SD	161.0 ± 10.2	161.3 ± 9.7	161.0 ± 10.2	0.85
Weight, kg, mean ± SD	59.7 ± 14.2	58.8 ± 14.2	59.8 ± 14.2	0.67
BMI, kg/m^2^, mean ± SD	22.9 ± 4.5	22.5 ± 4.5	23.0 ± 4.5	0.51
Emergency surgery, n (%)	336 (64.9)	33 (75.0)	303 (63.9)	0.14
Days from hospital admission to ICU admission				
Mean ± SD	7.3 ± 17.7	10.9 ± 31.5	7.0 ± 15.8	0.16
Median (IQR)	1.0 (0.0–6.0)	2.5 (0.0–7.3)	1.0 (0.0–6.0)	0.39
Chronic comorbidities, n (%)				
Heart failure	10 (1.9)	0	10 (2.1)	0.33
Respiratory failure	8 (1.5)	1 (2.3)	7 (1.5)	0.68
Liver cirrhosis	12 (2.3)	4 (9.1)	8 (1.7)	0.002
Acute leukemia	1 (0.2)	0	1 (0.2)	0.76
Lymphoma	5 (1.0)	0	5 (1.1)	0.49
Metastatic cancer	23 (4.4)	3 (6.8)	20 (4.2)	0.42
Immunosuppressive therapy	35 (6.8)	5 (11.4)	30 (6.3)	0.20
Maintenance dialysis	36 (6.9)	1 (2.3)	35 (7.4)	0.20
ICU interventions and prognostic indicators within 24 h of admission				
Mechanical ventilation, n (%)	386 (74.5)	44 (100.0)	342 (72.2)	< 0.001
Pulmonary artery catheterization, n (%)	24 (4.6)	11 (25.0)	13 (2.7)	< 0.001
Acute kidney injury, n (%)	46 (8.9)	5 (11.4)	41 (8.6)	0.54
Dopamine, n (%)	43 (8.3)	11 (25.0)	32 (6.8)	< 0.001
Missing	61 (11.8)	3 (6.8)	58 (12.2)	
Norepinephrine, n (%)	242 (46.7)	32 (72.7)	210 (44.3)	0.001
Missing	61 (11.8)	3 (6.8)	58 (12.2)	
Dobutamine, n (%)	67 (12.9)	11 (25.0)	56 (11.8)	0.03
Missing	61 (11.8)	3 (6.8)	58 (12.2)	
APACHE II, mean ± SD	24.5 ± 11.1	32.8 ± 9.1	23.7 ± 11.0	< 0.001
APACHE III, mean ± SD	94.0 ± 44.2	127.9 ± 35.5	90.9 ± 43.6	< 0.001
SAPS II, mean ± SD	53.9 ± 26.6	71.5 ± 19.4	52.2 ± 26.6	< 0.001
SOFA, mean ± SD	9.0 ± 4.8	12.6 ± 3.2	8.6 ± 4.8	< 0.001
Japan Risk of Death, mean ± SD	0.3 ± 0.3	0.5 ± 0.3	0.3 ± 0.3	< 0.001
Worst lactate, mean ± SD	6.1 ± 5.8	11.9 ± 6.8	5.6 ± 5.4	< 0.001

APACHE, Acute Physiologic and Chronic Health Evaluation; BMI, body mass index; CPR, cardiopulmonary resuscitation; ECPR, extracorporeal cardiopulmonary resuscitation; ICU, intensive care unit; IQR, interquartile range; SAPS, Simplified Acute Physiology Score; SOFA, Sequential Organ Failure Assessment.

Results are reported as odds ratios with corresponding 95% CIs. A two-sided *P* value less than 0.05 was considered statistically significant. All analyses were performed using R version 4.5.0 (R Foundation for Statistical Computing, Vienna, Austria).

## Results

**Fig. 1.**
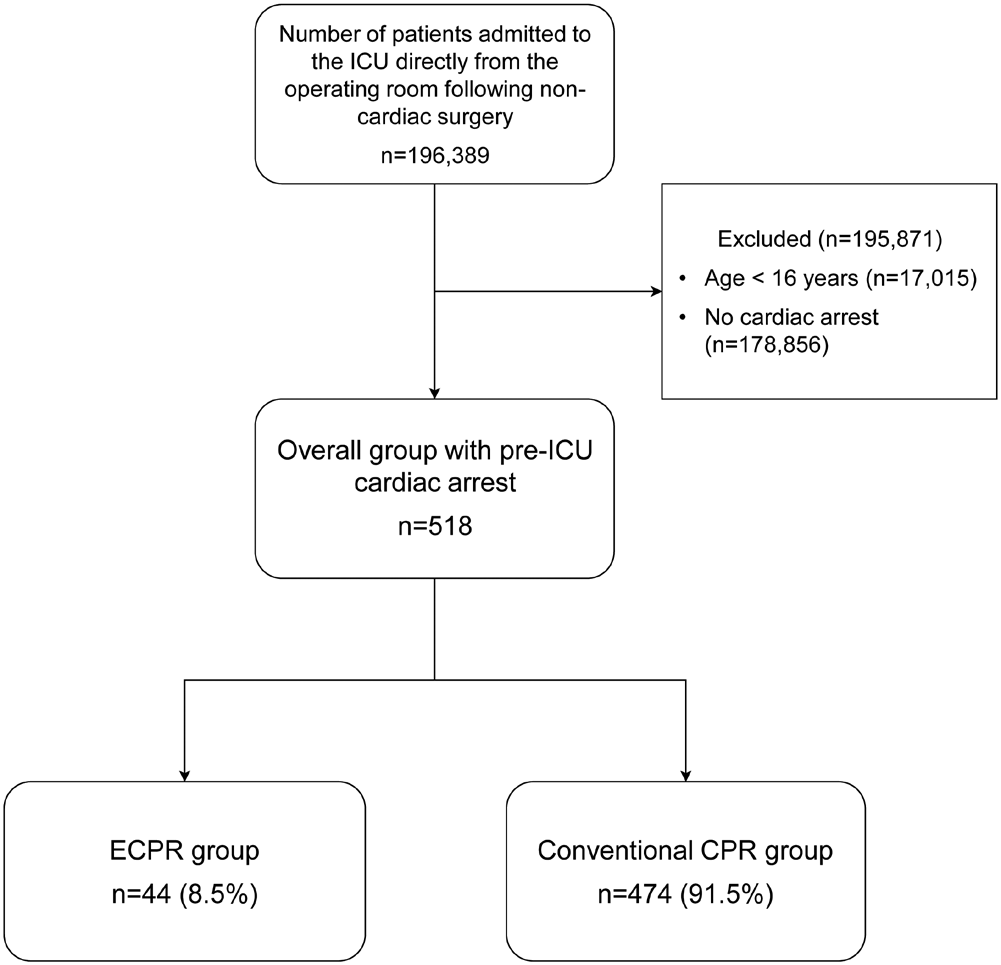
Flowchart of patient selection and group stratification. CPR, cardiopulmonary resuscitation; ECPR, extracorporeal cardiopulmonary resuscitation; ICU, intensive care unit.

During the study period, 196,389 patients were admitted to the ICU following noncardiac surgery. Of these, 195,871 met the predefined exclusion criteria and were excluded. The remaining 518 patients who experienced cardiac arrest before ICU admission were included in the analysis. Among them, 44 patients (8.5%) received ECPR and were classified as the ECPR group, while the remaining 474 patients (91.5%) did not receive ECPR and were classified as the conventional CPR group (fig. 1).

As shown in table 1, the mean age of the overall cohort was 66.0 yr and 319 of 518 patients (61.6%) were male. Emergency surgery was performed in 336 of 518 patients (64.9%). Compared with the conventional CPR group, patients in the ECPR group had a higher prevalence of liver cirrhosis and were more likely to receive mechanical ventilation, pulmonary artery catheterization, and catecholamines (dopamine, norepinephrine, and dobutamine). Severity scores—including APACHE II, APACHE III, Simplified Acute Physiology Score II, Sequential Organ Failure Assessment, and Japan Risk of Death—as well as lactate levels, were significantly higher in the ECPR group. Surgical diagnoses recorded at ICU admission are summarized in Supplemental Digital Content 2 (https://links.lww.com/ALNO/A7). The most frequent surgical admission diagnosis was “cardiac arrest” (Code 102: n = 69, 13.3%), a category denoting cardiac arrest before the initiation of the surgical procedure. Regarding specific surgical procedures, the most common indications were gastrointestinal neoplasm (n = 51, 9.8%), gastrointestinal perforation or rupture (n = 35, 6.8%), and multiple trauma excluding head (n = 32, 6.2%).

Of the 129 facilities participating in the JIPAD, 97 treated at least one patient with perioperative cardiac arrest. The characteristics of these 97 facilities, stratified by ECPR facilities (n = 33) and non-ECPR facilities (n = 64), are provided in Supplemental Digital Content 3 (https://links.lww.com/ALNO/A8). Compared with non-ECPR facilities, ECPR facilities had significantly higher numbers of total hospital beds, ICU beds, and full-time noncertified ICU physicians and nurses. To illustrate institutional heterogeneity, the facility-level distribution of case volume, ECPR implementation, and clinical outcomes across participating facilities is presented in Supplemental Digital Content 4 (https://links.lww.com/ALNO/A9). ECPR implementation rates ranged from 0 to 100%, with peak utilization observed in low-volume facilities.

Table 2 summarizes the clinical outcomes. In-hospital mortality was significantly higher in the ECPR group than in the conventional CPR group (61.4 *vs.* 34.0%; *P* = 0.001). Among the secondary outcomes, the ECPR group had a significantly lower rate of discharge to home (13.6 *vs.* 42.4%; *P* < 0.001) and a higher ICU mortality (52.3 *vs.* 23.2%; *P* < 0.001). There were no significant differences between groups regarding transfer to another healthcare facility, length of hospital stay, or length of ICU stay.

**Table 2.** Clinical Outcomes

Variable	Overall (N = 518)	ECPR (n = 44)	Conventional CPR (n = 474)	*P* Value
In-hospital mortality, n (%)	188 (36.3)	27 (61.4)	161 (34.0)	0.001
Discharge to home, n (%)	207 (40.0)	6 (13.6)	201 (42.4)	< 0.001
Transfer to another healthcare facility, n (%)	123 (23.7)	11 (25.0)	112 (23.6)	0.99
ICU mortality, n (%)	133 (25.7)	23 (52.3)	110 (23.2)	< 0.001
Length of hospital stay, median (IQR)	24.0 (10.0–55.0)	18.0 (7.8–47.3)	24.5 (10.0–55.0)	0.45
Length of ICU stay, median (IQR)	3.0 (1.0–7.0)	5.0 (1.0–12.3)	3.0 (1.0–7.0)	0.052

CPR, cardiopulmonary resuscitation; ECPR, extracorporeal cardiopulmonary resuscitation; ICU, intensive care unit; IQR, interquartile range.

**Fig. 2.**
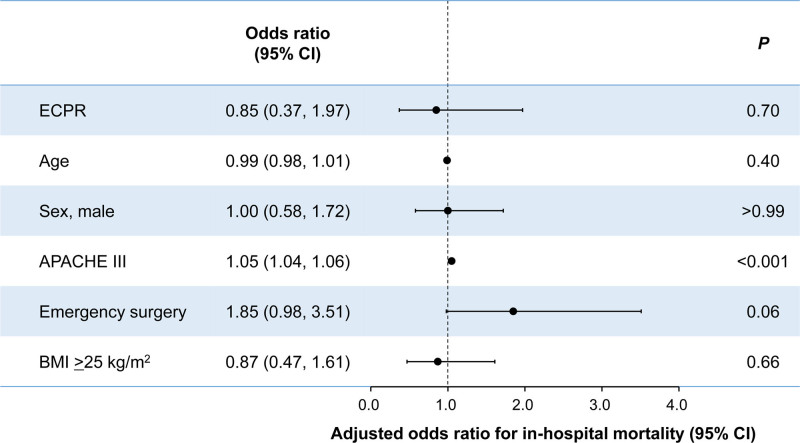
Multivariable analysis of factors associated with in-hospital mortality. Adjusted odds ratios with 95% CIs for in-hospital mortality were derived from the multivariable logistic regression using generalized estimating equations to account for patient clustering within facilities. APACHE, Acute Physiologic and Chronic Health Evaluation; BMI, body mass index; ECPR, extracorporeal cardiopulmonary resuscitation.

Figure 2 presents the results of the multivariable logistic regression analysis using Generalized Estimating Equations. In the primary analysis, adjusting for potential confounders, no statistically significant association was observed between ECPR and in-hospital mortality (odds ratio, 0.85; 95% CI, 0.37 to 1.97; *P* = 0.70). The lack of significant association remained consistent in three sensitivity analyses: backward selection, categorization of continuous covariates, and propensity score matching (table 3). After propensity score matching, baseline characteristics were generally balanced between the two groups (Supplemental Digital Content 5: https://links.lww.com/ALNO/A10).

**Table 3.** Sensitivity Analyses

	Odds Ratio	95% CI	*P* Value
Backward selection			
ECPR	0.81	0.36–1.83	0.61
APACHE III	1.06	1.05–1.06	< 0.001
Categorization of continuous covariates			
ECPR	1.30	0.61–2.76	0.49
Age ≥ 65 yr	1.29	0.76–2.17	0.35
Sex, male	1.07	0.67–1.70	0.78
APACHE III ≥ 89	20.30	10.79–38.20	< 0.001
Emergency surgery	1.83	1.00–3.33	0.049
BMI ≥ 25 kg/m^2^	0.83	0.47–1.45	0.51
Propensity score matching			
ECPR	1.17	0.39–3.60	0.78

Generalized estimating equations were used for multivariable regression models to account for facility-level clustering. Backward selection followed a *P* > 0.05 removal criterion. Categorization of continuous covariates involved dichotomizing age (< 65 *vs*. ≥ 65 yr) and the APACHE III score based on its median. Propensity score matching was performed at a 1:1 ratio.

APACHE, Acute Physiologic and Chronic Health Evaluation; BMI, body mass index; ECPR, extracorporeal cardiopulmonary resuscitation.

## Discussion

This multicenter retrospective cohort study, based on the JIPAD, evaluated the characteristics and outcomes of ECPR for perioperative cardiac arrest following noncardiac surgery. ECPR was performed in 44 of 518 patients (8.5%) who experienced cardiac arrest before ICU admission. Although unadjusted in-hospital mortality was significantly higher in the ECPR group than in the conventional CPR group, multivariable logistic regression using Generalized Estimating Equations demonstrated no significant association between ECPR and in-hospital mortality. This finding remained consistent across all sensitivity analyses. Ultimately, the effect of ECPR remains inconclusive due to the limited sample size and baseline imbalances; however, our data do not indicate that ECPR is more harmful than conventional CPR.

This study provides novel and generalizable insights by examining ECPR for perioperative cardiac arrest exclusively in noncardiac surgery using a nationwide ICU registry. Methodologically, we employed Generalized Estimating Equations to rigorously account for patient clustering within participating facilities. This approach mitigates the potential bias arising from institutional variations in practice and outcomes, ensuring more robust estimates than standard models that ignore the hierarchical structure of multicenter data. Few previous investigations have addressed this population because most available evidence derives from small, single-center case series with limited applicability.^13,25^ In cardiac surgery, VA-ECMO may be initiated either for refractory intraoperative cardiac arrest or for failure to wean from planned cardiopulmonary bypass. Because these scenarios are not always clearly distinguishable, published reports often include a heterogeneous mix of patients, making it difficult to assess the specific effect of ECPR.^26,27^

Our findings contrast with previous reports demonstrating survival benefits of ECPR in carefully selected patients with in-hospital cardiac arrest. Four factors may help explain this discrepancy. First, although in-hospital cardiac arrest arises from diverse causes, it has been extensively studied, incorporated into clinical practice guidelines, and managed under standardized protocols that emphasize rapid ECMO deployment and strict patient selection.^8,28,29^ By contrast, perioperative cardiac arrest occurs under highly specific intraoperative or anesthetic circumstances—such as airway complications, anesthetic-induced hemodynamic collapse, massive hemorrhage, or procedure-related injury—whose mechanisms and trajectories differ from conventional in-hospital cardiac arrest.^17,30–32^ Second, substantial confounding by indication is likely, as ECPR in perioperative settings is frequently attempted only after prolonged resuscitation without return of spontaneous circulation, suggesting that the intervention may have been initiated too late to reverse the underlying pathophysiology. Furthermore, in the context of noncardiac surgery, the potential benefits of ECPR might have been counterbalanced by associated harms, such as hemorrhagic complications exacerbated by systemic anticoagulation or cannulation-related injuries. In Japan, the absence of formal death pronouncement in the operating room may further encourage proceeding with ECMO initiation in these situations,^33^ resulting in the ECPR group disproportionately comprising patients with markedly limited physiological reserve. Third, even in out-of-hospital cardiac arrest—a population in which ECPR has been most rigorously evaluated—a recent randomized controlled trial demonstrated no significant improvement in survival with favorable neurological outcomes compared with conventional CPR,^34^ highlighting that the effectiveness of ECPR is not universal and may depend strongly on context and patient selection. Fourth, potential publication bias should be acknowledged because studies reporting poor outcomes of ECPR may be less likely to appear in the literature. Taken together, these factors may explain why no clear survival benefit of perioperative ECPR was observed in our cohort.

Notably, Japan has a well-established nationwide infrastructure for ECMO delivery, particularly in the setting of out-of-hospital cardiac arrest.^35,36^ However, perioperative ECPR in our study demonstrated only limited benefit. Its application should therefore be approached with even greater caution in countries with less organized or resource-constrained ECMO systems, where the balance between potential benefit and opportunity cost is especially critical. Ultimately, these findings highlight the ongoing challenge of determining how ECPR can be applied in a way that is both clinically justified and feasible across diverse healthcare systems.

Our results suggest that anesthesiologists should recognize the current limitations of ECPR in the perioperative setting and apply it selectively rather than routinely. Given the heterogeneity of perioperative cardiac arrest and the substantial resources required for ECMO deployment, international clinical practice guidelines recommend ECPR only in highly selected circumstances.^3–5^ For anesthesiologists—who often serve as the first responders and key decision-makers during perioperative cardiac arrest—this means prioritizing early, high-quality conventional resuscitation, while considering ECPR only when institutional resources, patient characteristics, and perioperative circumstances align to make a meaningful benefit plausible. In this role, anesthesiologists must carefully weigh both the immediate feasibility of ECPR and the balance between potential patient benefit and broader resource implications.

Several limitations should be considered when interpreting the results of this study. First, the definition of ECPR relied on VA-ECMO being recorded as an ICU treatment. Because JIPAD does not collect time-stamped information on ECMO initiation, the exact timing of cannulation cannot be determined, reflecting a design limitation of the registry rather than missing data. Second, the registry lacked standardized data on arrest etiologies. Although a qualitative review of text fields suggested a heterogeneous mix—ranging from reversible causes (*e.g.*, pulmonary embolism) to conditions with poor prognosis (*e.g.*, trauma, advanced malignancy)—formal analysis was not possible. Third, the registry lacked detailed arrest-related variables—including duration of cardiac arrest, time to initiation and duration of CPR, initial electrocardiographic rhythm, and low-flow duration for ECPR—leaving residual confounding from unmeasured prognostic factors.^22,37,38^ Fourth, functional outcome measures—such as neurological status or cerebral performance category—are not captured in the JIPAD, preventing any assessment of postarrest functional recovery. Fifth, although eligibility was restricted to noncardiac surgery, misclassification remains possible because code 102 (cardiac arrest without procedure) may have included patients who arrested while awaiting emergency cardiac surgery. Sixth, potential selection bias exists because ECPR was offered only to patients judged to have potentially salvageable conditions among those who did not achieve return of spontaneous circulation with conventional CPR. Patients who received ECPR therefore appeared more physiologically deranged at baseline, as reflected by higher severity scores and lactate levels—findings that align with the clinical context in which ECPR is considered, rather than contradicting its selective use. Seventh, the relatively small number of ECPR cases reduced statistical power and limited the ability to adjust for multiple covariates. Finally, the study was conducted in well-resourced Japanese ICUs participating in JIPAD, which may limit generalizability to other healthcare systems. Nonetheless, these limitations are unlikely to alter the overall interpretation because this study provides the largest multicenter analysis to date of ECPR for perioperative cardiac arrest after noncardiac surgery.

In conclusion, this multicenter retrospective cohort study demonstrated that ECPR was performed in 8.5% of patients with perioperative cardiac arrest during noncardiac surgery. The effect of ECPR on in-hospital mortality in this population remains inconclusive, primarily due to the limited sample size and baseline imbalances. However, our data do not indicate that ECPR is more harmful than conventional CPR. Continued cautious use in current practice will allow the accumulation of additional data to better define its effect on outcomes.

## Acknowledgments

The authors thank Angela Morben, D.V.M., E.L.S., from Edanz (https://jp.edanz.com/ac, Fukuoka, Japan), for editing a draft of this manuscript. During the preparation of this manuscript, the authors used ChatGPT (GPT-5, OpenAI) to assist with improving the readability and language clarity of the English text. The AI tool was not used for data analysis, content generation, or scientific interpretation. All authors have critically reviewed and revised the AI-assisted text and accept full responsibility for the integrity and accuracy of the content.

## Research Support

This work was supported by JSPS KAKENHI Grant Number JP22K09044 and institutional sources from Hamamatsu University School of Medicine.

## Competing Interests

The authors declare no competing interests.

## Supplemental Digital Content

Diagnosis codes for noncardiac surgery, Supplemental Digital Content 1: https://links.lww.com/ALNO/A6.

Surgical diagnoses at intensive care admission, Supplemental Digital Content 2: https://links.lww.com/ALNO/A7.

Characteristics of participating facilities, Supplemental Digital Content 3: https://links.lww.com/ALNO/A8.

Facility extracorporeal resuscitation volume, Supplemental Digital Content 4: https://links.lww.com/ALNO/A9.

Characteristics before and after matching, Supplemental Digital Content 5: https://links.lww.com/ALNO/A10.

## Supplementary Material
