# Supplementary material for: Extracorporeal Cardiopulmonary Resuscitation for Perioperative Cardiac Arrest in Noncardiac Surgery: A Nationwide Cohort Study in Japan
Source: Anesthesiol Open. 2026 Apr 15;1(1):e0013. doi: 10.1097/ao9.0000000000000013 (PMC13086117; doi:10.1097/ao9.0000000000000013)
Supplement: Supplementary file 1 [file ao9-1-e0013-s001.pdf]

Supplemental Digital Content 1. Diagnosis codes for noncardiac surgery

Respiratory

1301. Respiratory infection
1302. Respiratory neoplasm – lung
1303. Respiratory neoplasm – mouth, larynx, sinus, trachea
1304. Other respiratory diseases

Gastrointestinal (GI)

1401. GI perforation/rupture (not peritonitis)
1403. GI bleeding
1404. GI obstruction
1405. GI neoplasm
1406. Cholecystitis/Cholangitis
1407. Liver transplant
1408. Other GI diseases
1409. Fistula/Abscess surgery
1410. GI vascular ischemia resection surgery
1411. Pancreatitis
1412. Peritonitis
1413. Other GI inflammatory disease

Neurological

1501. Intracerebral hemorrhage
1502. Subdural/Epidural hematoma
1503. Subarachnoid hemorrhage
1504. Laminectomy/Spinal cord surgery
1505. Craniotomy for neoplasm
1506. Other neurologic disease
1507. Unruptured aneurysm clipping
1508. Unruptured aneurysm coil embolization
1509. Cerebral infarction

Trauma

1601. Head trauma +/- multi trauma
1602. Multiple traumas excluding head

1603. Burns
1604. Multiple traumas with spinal injury
1605. Isolated cervical spine injury

Renal/Genitourinary
1701. Renal neoplasm
1703. Other renal diseases
1704. Kidney transplant
1705. Genitourinary surgery/procedure

Gynecological
1801. Hysterectomy
1802. Pregnancy-related disorder
1803. Other gynecological disease

Musculoskeletal
1902. Orthopedic surgery
1903. Skin surgery
1904. Cellulitis/Soft tissue infection

Hematological
2101. Hematological disease

Metabolic
2201. Metabolic disease

Cardiovascular
102. Cardiac arrest
*This code indicates that the patient was admitted to the ICU due to cardiac arrest without having undergone any surgical procedures.
