# Supplementary material for: Extracorporeal Cardiopulmonary Resuscitation for Perioperative Cardiac Arrest in Noncardiac Surgery: A Nationwide Cohort Study in Japan
Source: Anesthesiol Open. 2026 Apr 15;1(1):e0013. doi: 10.1097/ao9.0000000000000013 (PMC13086117; doi:10.1097/ao9.0000000000000013)
Supplement: Supplementary file 2 [file ao9-1-e0013-s002.pdf]

Supplemental Digital Content 2. Surgical diagnoses at intensive care admission

Overall				ECPR				Conventional CPR			
Code	Surgery	n	%	Code	Surgery	n	%	Code	Surgery	n	%
102	Cardiac arrest	69	13.3	102	Cardiac arrest	14	31.8	102	Cardiac arrest	55	11.6
1405	GI neoplasm	51	9.8	1405	GI neoplasm	6	13.6	1405	GI neoplasm	45	9.5
1401	GI perforation/rupture (not peritonitis)	35	6.8	1401	GI perforation/rupture (not peritonitis)	3	6.8	1401	GI perforation/rupture (not peritonitis)	32	6.8
1602	Multiple trauma excluding head	32	6.2	1304	Other respiratory diseases	2	4.5	1602	Multiple trauma excluding head	30	6.3
1902	Orthopedic surgery	29	5.6	1408	Other GI diseases	2	4.5	1902	Orthopedic surgery	27	5.7
1302	Respiratory neoplasm – lung	27	5.2	1602	Multiple trauma excluding head	2	4.5	1302	Respiratory neoplasm – lung	26	5.5
1503	Subarachnoid hemorrhage	20	3.9	1802	Pregnancy-related disorder	2	4.5	1503	Subarachnoid hemorrhage	20	4.2
1304	Other respiratory diseases	19	3.7	1902	Orthopedic surgery	2	4.5	1304	Other respiratory diseases	17	3.6
1601	Head trauma +/- multi trauma	17	3.3	1302	Respiratory neoplasm – lung	1	2.3	1601	Head trauma +/- multi trauma	17	3.6
1410	GI vascular ischemia resection surgery	16	3.1	1403	GI bleeding	1	2.3	1404	GI obstruction	15	3.2
1404	GI obstruction	15	2.9	1406	Cholecystitis/Cholangitis	1	2.3	1410	GI vascular ischemia resection surgery	15	3.2
1408	Other GI diseases	13	2.5	1407	Liver transplant	1	2.3	1403	GI bleeding	11	2.3
1403	GI bleeding	12	2.3	1410	GI vascular ischemia resection surgery	1	2.3	1408	Other GI diseases	11	2.3
1903	Skin surgery	11	2.1	1413	Other GI inflammatory disease	1	2.3	1903	Skin surgery	11	2.3
1501	Intracerebral hemorrhage	10	1.9	1701	Renal neoplasm	1	2.3	1501	Intracerebral hemorrhage	10	2.1
1505	Craniotomy for neoplasm	10	1.9	1801	Hysterectomy	1	2.3	1505	Craniotomy for neoplasm	10	2.1

CPR, cardiopulmonary resuscitation; ECPR, extracorporeal cardiopulmonary resuscitation; GI, Gastrointestinal; ICU, intensive care unit.
