# Supplementary material for: Extracorporeal Cardiopulmonary Resuscitation for Perioperative Cardiac Arrest in Noncardiac Surgery: A Nationwide Cohort Study in Japan
Source: Anesthesiol Open. 2026 Apr 15;1(1):e0013. doi: 10.1097/ao9.0000000000000013 (PMC13086117; doi:10.1097/ao9.0000000000000013)
Supplement: Supplementary file 3 [file ao9-1-e0013-s003.pdf]

Supplemental Digital Content 3. Characteristics of participating facilities

	Overall (n=97)*	ECPR facilities (n=33)*	Non-ECPR facilities (n=64)	*p*
Hospital type, n (%)				0.54
National university hospital	19 (19.8)	8 (25.0)	11 (17.2)	
National hospital	9 (9.4)	1 (3.1)	8 (12.5)	
Private university hospital	14 (14.6)	6 (18.8)	8 (12.5)	
Private hospital	17 (17.7)	4 (12.5)	13 (20.3)	
Public university hospital	5 (5.2)	2 (6.3)	3 (4.7)	
Public hospital	32 (33.3)	11 (34.4)	21 (32.8)	
ICU functional type, n (%)				0.75
Emergency and Medical-Surgical ICU	47 (49.0)	15 (46.9)	32 (50.0)	
Medical-Surgical ICU	30 (31.3)	11 (34.4)	19 (29.7)	
Emergency ICU	9 (9.4)	4 (12.5)	5 (7.8)	
Surgical ICU	8 (8.3)	2 (6.3)	6 (9.4)	
Other units	2 (2.1)	0 (0.0)	2 (3.1)	
ICU operational model, n (%)				>0.99
Mandatory critical care consultation	57 (59.4)	19 (59.4)	38 (59.4)	
Closed ICU	21 (21.9)	7 (21.9)	14 (21.9)	
Elective critical care consultation	18 (18.8)	6 (18.8)	12 (18.8)	
Total number of hospital beds, mean (SD)	704.0 (249.8)	784.6 (269.0)	663.7 (231.3)	0.02
Number of ICU beds, mean (SD)	12.5 (5.8)	14.3 (6.1)	11.6 (5.5)	0.03
Number of full-time ICU staff, mean (SD)				
ICU physician certified by the JSICM	2.8 (2.1)	3.3 (2.2)	2.5 (2.0)	0.11
Non-certified ICU physician	3.7 (4.8)	5.1 (5.9)	3.0 (4.0)	0.04
Nurse	43.3 (23.8)	52.1 (31.4)	38.9 (17.5)	0.01

ICU, intensive care unit; SD, standard deviation; JSICM, Japanese Society of Intensive Care Medicine.

*Data on facility characteristics were not available for one institution because the information was missing in the JIPAD. Therefore, this table presents data for 96 facilities (32 ECPR facilities and 64 non-ECPR facilities).
