# Supplementary material for: Extracorporeal Cardiopulmonary Resuscitation for Perioperative Cardiac Arrest in Noncardiac Surgery: A Nationwide Cohort Study in Japan
Source: Anesthesiol Open. 2026 Apr 15;1(1):e0013. doi: 10.1097/ao9.0000000000000013 (PMC13086117; doi:10.1097/ao9.0000000000000013)
Supplement: Supplementary file 4 [file ao9-1-e0013-s004.pdf]

Supplemental Digital Content 4. Facility extracorporeal resuscitation volume

Total Perioperative Cardiac Arrests, n	Total non-survivors, n (%)	ECPR Implementation, n (%)	ECPR non-survivors, n (%)
22	6 (27.3)	1 (4.5)	0 (0.0)
19	5 (26.3)	0 (0.0)	-
15	8 (53.3)	1 (6.7)	1 (100.0)
15	2 (13.3)	0 (0.0)	-
13	3 (23.1)	3 (23.1)	2 (66.7)
13	5 (38.5)	1 (7.7)	1 (100.0)
13	4 (30.8)	0 (0.0)	-
13	8 (61.5)	1 (7.7)	1 (100.0)
13	0 (0.0)	0 (0.0)	-
12	7 (58.3)	0 (0.0)	-
12	7 (58.3)	1 (8.3)	0 (0.0)
12	5 (41.7)	4 (33.3)	2 (50.0)
11	7 (63.6)	2 (18.2)	2 (100.0)
11	8 (72.7)	0 (0.0)	-
11	1 (9.1)	1 (9.1)	1 (100.0)
10	2 (20.0)	2 (20.0)	0 (0.0)
10	4 (40.0)	1 (10.0)	1 (100.0)
10	0 (0.0)	1 (10.0)	0 (0.0)
10	4 (40.0)	1 (10.0)	1 (100.0)
9	3 (33.3)	1 (11.1)	1 (100.0)
9	3 (33.3)	1 (11.1)	1 (100.0)
9	3 (33.3)	0 (0.0)	-
9	1 (11.1)	0 (0.0)	-
9	3 (33.3)	3 (33.3)	2 (66.7)
9	5 (55.6)	1 (11.1)	1 (100.0)
8	4 (50.0)	0 (0.0)	-
8	5 (62.5)	1 (12.5)	1 (100.0)
8	1 (12.5)	0 (0.0)	-
8	1 (12.5)	0 (0.0)	-
8	5 (62.5)	0 (0.0)	-
8	2 (25.0)	0 (0.0)	-
7	6 (85.7)	0 (0.0)	-
7	1 (14.3)	0 (0.0)	-
6	2 (33.3)	0 (0.0)	-
6	5 (83.3)	0 (0.0)	-
5	2 (40.0)	0 (0.0)	-
5	0 (0.0)	1 (20.0)	0 (0.0)
5	1 (20.0)	0 (0.0)	-
5	2 (40.0)	1 (20.0)	1 (100.0)
5	2 (40.0)	0 (0.0)	-
5	3 (60.0)	2 (40.0)	2 (100.0)
4	1 (25.0)	0 (0.0)	-
4	2 (50.0)	1 (25.0)	0 (0.0)
4	4 (100.0)	0 (0.0)	-
4	0 (0.0)	0 (0.0)	-
3	0 (0.0)	0 (0.0)	-
3	2 (66.7)	0 (0.0)	-

3	2 (66.7)	1 (33.3)	0 (0.0)
3	1 (33.3)	0 (0.0)	-
3	1 (33.3)	2 (66.7)	1 (50.0)
3	1 (33.3)	0 (0.0)	-
3	2 (66.7)	1 (33.3)	0 (0.0)
3	1 (33.3)	0 (0.0)	-
3	0 (0.0)	1 (33.3)	0 (0.0)
3	0 (0.0)	0 (0.0)	-
3	2 (66.7)	0 (0.0)	-
3	0 (0.0)	0 (0.0)	-
3	2 (66.7)	0 (0.0)	-
3	1 (33.3)	1 (33.3)	0 (0.0)
2	1 (50.0)	0 (0.0)	-
2	0 (0.0)	0 (0.0)	-
2	0 (0.0)	0 (0.0)	-
2	0 (0.0)	1 (50.0)	0 (0.0)
2	2 (100.0)	0 (0.0)	-
2	0 (0.0)	0 (0.0)	-
2	2 (100.0)	1 (50.0)	1 (100.0)
2	1 (50.0)	0 (0.0)	-
2	0 (0.0)	0 (0.0)	-
2	1 (50.0)	0 (0.0)	-
2	1 (50.0)	0 (0.0)	-
2	2 (100.0)	1 (50.0)	1 (100.0)
2	2 (100.0)	1 (50.0)	1 (100.0)
2	0 (0.0)	0 (0.0)	-
2	0 (0.0)	0 (0.0)	-
2	0 (0.0)	0 (0.0)	-
2	2 (100.0)	0 (0.0)	-
2	1 (50.0)	0 (0.0)	-
2	1 (50.0)	1 (50.0)	1 (100.0)
1	1 (100.0)	0 (0.0)	-
1	1 (100.0)	0 (0.0)	-
1	0 (0.0)	0 (0.0)	-
1	0 (0.0)	0 (0.0)	-
1	0 (0.0)	0 (0.0)	-
1	0 (0.0)	0 (0.0)	-
1	0 (0.0)	0 (0.0)	-
1	0 (0.0)	0 (0.0)	-
1	0 (0.0)	0 (0.0)	-
1	0 (0.0)	0 (0.0)	-
1	0 (0.0)	0 (0.0)	-
1	0 (0.0)	0 (0.0)	-
1	0 (0.0)	0 (0.0)	-
1	0 (0.0)	0 (0.0)	-
1	0 (0.0)	0 (0.0)	-
1	1 (100.0)	1 (100.0)	1 (100.0)
1	0 (0.0)	0 (0.0)	-
1	0 (0.0)	0 (0.0)	-
1	1 (100.0)	0 (0.0)	-

ECPR, extracorporeal cardiopulmonary resuscitation.
