# Supplementary material for: Extracorporeal Cardiopulmonary Resuscitation for Perioperative Cardiac Arrest in Noncardiac Surgery: A Nationwide Cohort Study in Japan
Source: Anesthesiol Open. 2026 Apr 15;1(1):e0013. doi: 10.1097/ao9.0000000000000013 (PMC13086117; doi:10.1097/ao9.0000000000000013)
Supplement: Supplementary file 5 [file ao9-1-e0013-s005.pdf]

Supplemental Digital Content 5. Characteristics before and after matching

Variable	Before propensity score matching			After propensity score matching		
	ECPR (n=44)	Conventional CPR (n=474)	Absolute standardized differences (%)	ECPR (n=26)	Conventional CPR (n=26)	Absolute standardized differences (%)
Age (year), mean (SD)	62.8 (17.2)	66.3 (16.5)	20.9	64.3 (15.6)	61.3 (19.8)	17.1
Male sex, n (%)	25 (56.8)	294 (62.0)	10.6	17 (65.4)	14 (53.8)	23.7
BMI (kg/m^2), mean (SD)	22.5 (4.5)	23.0 (4.5)	10.5	22.7 (4.8)	21.1 (4.2)	35.7
Emergency surgery, n (%)	33 (75.0)	303 (63.9)	60.4	21 (80.8)	21 (80.8)	<0.1
Days from hospital admission to ICU admission, mean (SD)	10.9 (31.5)	7.0 (15.8)	15.7	7.3 (13.4)	6.2 (9.6)	9.2
Chronic comorbidities, n (%)						
Heart failure	0	10 (2.1)	20.8	0	0	<0.1
Respiratory failure	1 (2.3)	7 (1.5)	5.9	0	0	<0.1
Liver cirrhosis	4 (9.1)	8 (1.7)	33.2	1 (3.8)	2 (7.7)	16.6
Acute leukemia	0	1 (0.2)	6.5	0	0	<0.1
Lymphoma	0	5 (1.1)	14.6	0	0	<0.1
Metastatic cancer	3 (6.8)	20 (4.2)	11.4	2 (7.7)	3 (11.5)	13.1
Immunosuppressive therapy	5 (11.4)	30 (6.3)	17.8	3 (11.5)	2 (7.7)	13.1
Maintenance dialysis	1 (2.3)	35 (7.4)	24.0	1 (3.8)	1 (3.8)	<0.1
**ICU interventions and prognostic indicators within 24 hours of admission**						
Mechanical ventilation, n (%)	44 (100.0)	342 (72.2)	87.9	26 (100.0)	26 (100.0)	<0.1
Pulmonary artery catheterization, n (%)	11 (25.0)	13 (2.7)	68.0	7 (26.9)	4 (15.4)	28.5
Acute kidney injury, n (%)	5 (11.4)	41 (8.6)	9.1	4 (15.4)	1 (3.8)	39.9
Dopamine, n (%)	11 (25.0)	32 (6.8)	53.0	8 (30.8)	6 (23.1)	17.4
Norepinephrine, n (%)	32 (72.7)	210 (44.3)	60.4	22 (84.6)	22 (84.6)	<0.1
Dobutamine, n (%)	3 (6.8)	58 (12.2)	37.2	6 (23.1)	7 (26.9)	8.9
APACHE II, mean (SD)	32.8 (9.1)	23.7 (11.0)	90.3	31.4 (10.1)	32.1 (10.4)	6.8
APACHE III, mean (SD)	127.9 (35.5)	90.9 (43.6)	93.0	124.3 (39.4)	123.1 (41.7)	2.9
SAPS II, mean (SD)	71.5 (19.4)	52.2 (26.6)	82.6	70.3 (22.0)	68.9 (23.6)	6.2
SOFA, mean (SD)	12.6 (3.2)	8.6 (4.8)	97.7	12.4 (3.7)	11.7 (3.8)	18.6
JROD, mean (SD)	0.5 (0.3)	0.3 (0.3)	84.6	0.5 (0.3)	0.5 (0.3)	9.4
Worst lactate, mean (SD)	11.9 (6.8)	5.6 (5.4)	102.8	12.0 (6.8)	11.9 (7.1)	1.5

APACHE, Acute Physiologic and Chronic Health Evaluation; BMI, body mass index; CPR, cardiopulmonary resuscitation; ECPR, extracorporeal cardiopulmonary resuscitation; ICU, intensive care unit; JROD, Japan Risk of Death; SAPS, Simplified Acute Physiology Score; SD, standard deviation; SOFA, Sequential Organ Failure Assessment.
Note: In the propensity score-matched cohort (26 pairs), in-hospital mortality was 61.5% (16/26) in the ECPR group and 57.7% (15/26) in the conventional CPR group.
